# The Spatiotemporal Distribution Patterns and Coexistence Mechanisms of Two Musk Deer Species in Mt. Gongga, Sichuan Province, China

**DOI:** 10.3390/ani15182701

**Published:** 2025-09-15

**Authors:** Nan Yang, Yuanhao Du, Mingyang Liu, Yong Jiang, Xingcheng He

**Affiliations:** 1 College of Grassland Resources, Southwest Minzu University, Chengdu 610041, China; yangnan0204@126.com (N.Y.); lmychem@163.com (M.L.); 2 Sichuan Provincial Forest and Grassland Key Laboratory of Alpine Grassland Conservation and Utilization of Qinghai–Tibet Plateau, Chengdu 610041, China; 3 Key Laboratory of Bio-Resources and Eco-Environment, Ministry of Education, College of Life Science, Sichuan University, Chengdu 610065, China; 2022222040131@stu.scu.edu.cn; 4 The Conservation of Endangered Wildlife Key Laboratory of Sichuan Province, Chengdu 610050, China; 5 Gongga Mountain National Nature Reserve Administration Bureau, Kangding 626000, China; jiangyong_1206@126.com

**Keywords:** Gongga Mountain National Nature Reserve, forest musk deer, alpine musk deer, closely related sympatric species, sympatric coexistence, ecological niche differentiation

## Abstract

Elucidating the spatiotemporal distribution patterns of closely related sympatric species is essential for understanding niche partitioning dynamics. The forest musk deer (*Moschus berezovskii*) and alpine musk deer (*Moschus chrysogaster*) extensively co-occur across the southeastern Qinghai–Tibet Plateau, yet their coexistence mechanisms remain unclear. Based on a decade-long dataset (2012–2021) collected from 493 infrared cameras deployed in Gongga Mountain National Nature Reserve, this study integrated MaxEnt modeling, kernel density estimation, and niche overlap analysis to investigate their spatiotemporal distribution patterns. Spatially, the two species exhibited strong geographic consistency and similar ecological preferences for climate and vegetation factors. Both species shared an elevational range (2870–4572 m ASL) with no clear segregation, likely due to climate-driven forest line shifts. In contrast, temporal partitioning was pronounced: *M. berezovskii* was mostly nocturnal, while *M. chrysogaster* was primarily diurnal, with both showing seasonal activity adjustments. These findings highlight temporal niche differentiation as a key coexistence strategy and emphasize the necessity of incorporating geographic variation in habitat adaptation into conservation frameworks.

## Simple Summary

Forest musk deer (*Moschus berezovskii*) and alpine musk deer (*Moschus chrysogaster*), both classified as China’s National Grade I Protected Wildlife, coexist in the southeastern mountainous regions of the Qinghai–Tibet Plateau. Alpine musk deer were once thought to occupy higher-elevation shrubland and meadow habitats to reduce competition with forest musk deer. Nevertheless, a decade of camera-trapping data from Gongga Mountain National Nature Reserve, where the forest line extends up to 3830 m ASL, reveals that both species occupy overlapping high-altitude habitats (2870–4572 m ASL) without evidence of spatial segregation. Crucially, they avoid conflict through temporal partitioning: forest musk deer are primarily nocturnal, while alpine musk deer are mainly diurnal, with both showing seasonal activity adjustments. This “time-sharing” strategy enables stable coexistence. Our findings demonstrate that conservation strategies must account for species-specific adaptations to local geography and climate.

## 1. Introduction

Understanding how ecologically similar sympatric species achieve stable coexistence remains one of the main goals in community ecology [1]. According to theoretical frameworks, coexistence requires differentiation along at least one niche axis (spatial, temporal, or trophic) to reduce interspecific competition [2]. Ungulates, as key ecosystem engineers, significantly influence forest and grassland dynamics through grazing, trampling, and seed dispersal [3,4]. However, research on niche partitioning and coexistence mechanisms among sympatric ungulates has predominantly focused on large- and medium-sized species, leaving the ecological strategies of smaller and often more vulnerable taxa comparatively unexplored.

The forest musk deer (*Moschus. berezovskii* Flerov, 1929) and alpine musk deer (*M. chrysogaster* Hodgson, 1839) (Artiodactyla: Moschidae) are solitary, forest-dependent ungulates, both designated as Class I nationally protected species in China. *Moschus berezovskii* currently maintains a fragmented distribution across western Sichuan, southern Shaanxi, and eastern Tibet [5,6], while *M. chrysogaster*, a high-altitude specialist shaped by the uplift of the Qinghai–Tibet Plateau, is restricted to alpine and subalpine habitats within and around the plateau [5]. Musk deer populations have experienced catastrophic declines under sustained poaching pressure, driven by the commercial demand for musk in perfume production and traditional medicine. A national survey in 2003 indicated that musk deer populations in China had declined to only 2–3% of their levels in the 1950s, with the *M. berezovskii* numbering about 100,000–200,000 individuals in the wild and the *M. chrysogaster* about 100,000 [7,8], emphasizing the need for ecological studies to inform conservation strategies.

The southeastern Qinghai–Tibet Plateau forms a key sympatric region for the endangered forest musk deer and alpine musk deer. Although previous studies have identified their individual habitat preferences [9,10,11,12], the extent to which spatiotemporal niche partitioning mediates their coexistence remains largely unresolved within this biodiversity-rich region. Gongga Mountain National Nature Reserve (29°20′–30°20′ N, 101°30′–102°15′ E), situated within the ecologically complex Hengduan Mountains, provides a rare context for examining sympatric interactions between these species under protected conditions. Based on ecological niche theory, we formulated two complementary hypotheses regarding the coexistence of *Moschus berezovskii* and *M. chrysogaster*. H1 (Spatial niche hypothesis): the two species exhibit substantial spatial overlap in distribution but differ in microhabitat selection as a mechanism facilitating coexistence. H2 (Temporal niche hypothesis): given the high degree of spatial niche overlap, temporal niche partitioning serves as the primary coexistence mechanism, with the two species displaying distinct diel activity patterns.

## 2. Materials and Methods

### 2.1. Study Area

Gongga Mountain National Nature Reserve (101°29′–102°12′ E and 29°01′–30°05′ N) is located in the central segment of the Daxue Mountains, part of the Hengduan Mountain system on the southeastern margin of the Qinghai–Tibet Plateau. Recognized as a national biodiversity conservation priority area (http://www.mee.gov.cn) and a core region within a global biodiversity hotspot (https://www.conservation.org), the reserve covers a total area of 4091.4 km^2^, with its main peak reaching an elevation of 7508.9 m ASL. Conservation targets focus on preserving montane forest ecosystems centered around Gongga Mountain, protecting low-altitude modern glaciers, and safeguarding rare and endangered wildlife and plant species (Master Plan for Sichuan Gongga Mountain National Nature Reserve, 2019–2030). The reserve features a temperate plateau climate, structured into seven distinct climatic zones along its altitudinal gradient: subtropical, warm-temperate, cold-temperate, subarctic, arctic, frigid, and perpetual snow zones. Mt. Gongga’s vegetation changes sharply with altitude, ranging from degraded lowland shrub–grass, agroforestry, and broadleaf forests below 2000 m ASL, through a series of mixed broadleaf and coniferous forests, shrub–grass belts, and meadows between 2000 and 4900 m ASL, to permafrost above 4900 m ASL [13]. Unexpectedly, the unique climatic conditions of Gongga Mountain have caused its tree line to reach an altitude of 3830 m ASL [14]. The resulting environmental heterogeneity supports a wide range of ecosystem types, providing critical habitats for diverse wildlife assemblages. Mt. Gongga hosts numerous carnivores, including *Catopuma temminckii*, *Felis bieti*, *Otocolobus manul*, *Prionailurus bengalensis*, *Panthera pardus*, *Panthera uncia*, *Paguma larvata*, *Canis lupus*, *Vulpes vulpes*, and *Martes flavigula*. In addition to *M. berezovskii* and *M. chrysogaster*, the area also supports several other ungulate species that may compete for resources, such as *Rusa unicolor*, *Elaphodus cephalophus*, *Muntiacus vaginalis*, *Budorcas taxicolor*, *Capricornis milneedwardsii*, *Naemorhedus griseus*, and *Pseudois nayaur* [15].

### 2.2. Species Data Source and Processing

From 2012 to 2021, a systematic camera-trapping survey was implemented across Gongga Mountain National Nature Reserve and adjacent areas, resulting in 493 independent camera deployment sites. The survey accumulated a total of 102,879 camera-trap days, providing extensive spatial and temporal coverage for wildlife monitoring.

Camera sites were strategically selected to ensure representative habitat coverage, accounting for (1) habitat type distribution, (2) wildlife occurrence probability, and (3) field accessibility. Historical survey records from the reserve were integrated to prioritize areas with evidence of high wildlife activity, including game trails and scat presence. Cameras were deployed without bait near natural wildlife trails and water sources, and minor vegetation trimming was conducted where necessary to optimize detection probability while preserving animal movement corridors. All units operated continuously with photo-plus-video settings (three still images and a 15–20 s video per trigger event), set at medium to high sensitivity and maximum resolution (typically 2560 × 1920 pixels). This setting was adopted to maximize the amount of wildlife information collected while balancing the constraints of limited memory capacity.

**Figure 1:**
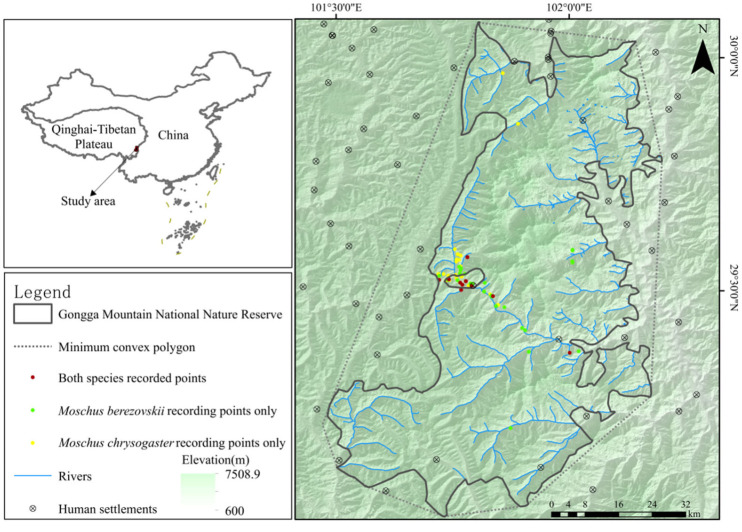
Occurrence locations of *Moschus berezovskii* and *Moschus chrysogaster* in Gongga Mountain National Nature Reserve.

To minimize spatial autocorrelation and reduce overfitting in subsequent modeling, spatial filtering was applied based on an estimated annual home range of approximately 0.3 km^2^ for both species [16]. Using ArcGIS 10.7, a 300 m radius buffer (0.28 km^2^) was generated around each detection point. When multiple conspecific records occurred within the same buffer zone, one record was randomly selected to represent that area. Following spatial thinning, 32 forest musk deer and 31 alpine musk deer records were retained for subsequent habitat suitability analysis (Figure 1).

### 2.3. Environmental Data Source and Processing

Ungulate habitat selection is largely shaped by environmental variables, particularly climate, topography, vegetation structure, and proximity to water sources [17,18,19]. In addition, shifts in human land-use practices have increasingly introduced anthropogenic disturbance as a major determinant of wildlife distribution patterns [20]. Based on these considerations, 24 environmental variables were selected, representing five major categories (climate (19), topography (2), vegetation (2), water sources (1), and human disturbance (1)) as potential predictors of habitat suitability for *M. berezovskii* and *M. chrysogaster.* Since the 19 bioclimatic variables are all derived from temperature and precipitation using different algorithms, and to avoid the negative effects of multicollinearity on model predictions, they were pre-screened using the MaxEnt model and ENMTools 1.4. Only variables with a contribution rate greater than 1% and a correlation coefficient < |0.75| were retained [21]. Ultimately, four bioclimatic variables (mean diurnal range, temperature annual range, precipitation seasonality, and precipitation of the driest quarter) were selected for model construction (Table 1). Nine vegetation types were defined: agricultural land, aquatic area, broadleaf forest, coniferous forest, meadow, mixed coniferous–broadleaf forest, scree, shrubland, and woodland grassland.

### 2.4. MaxEnt Modeling

Environmental predictors were assigned as categorical (e.g., vegetation type) or continuous variables according to their data structure. Model stability was evaluated through 10 bootstrap replicates, and logistic outputs were averaged across runs to generate final predictions [21]. Model performance was assessed using the area under the receiver operating characteristic curve (AUC), with values ranging from 0 to 1 [22]. Both *M. berezovskii* and *M. chrysogaster* models demonstrated robust predictive performance, with training and test AUC values exceeding 0.90 across replicates. The standard deviations (SD) were low (0.041 and 0.025, respectively), indicating high model reliability for habitat suitability estimation.

The balance training omission, predicted area, and threshold value (TPT) from the 10 MaxEnt replicates [23] were applied in ArcGIS v10.7 to binarize habitat suitability outputs. Binary maps for each species were subsequently overlaid through arithmetic addition to identify suitable overlapping habitats.

Ecological niche overlap between *M. berezovskii* and *M. chrysogaster* was assessed using Hellinger’s I index (ranging from 0 to 1) implemented in ENMTools v1.4 [24], with higher values indicating greater habitat similarity between species.

### 2.5. Kernel Density Estimation

**Figure 2:**
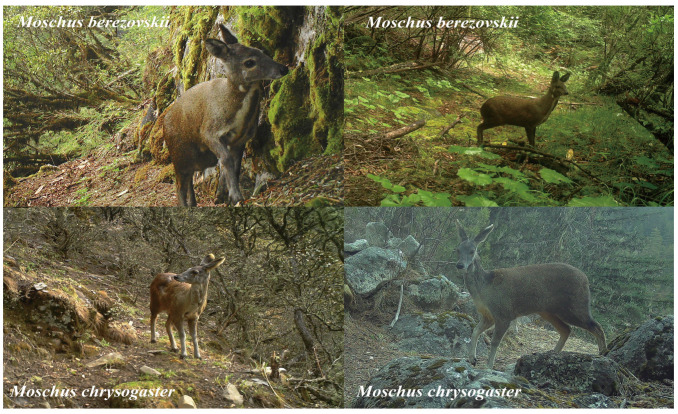
Infrared camera photos of *Moschus berezovskii* and *Moschus chrysogaster* in Gongga Mountain National Nature Reserve.

Temporal activity patterns were assessed using 10 years (2012–2021) of infrared camera-trap data collected from Gongga Mountain National Nature Reserve. To minimize pseudo-replication, independent detection events were defined using a 30 min interval between consecutive records of the same species at the same site. After filtering, 103 valid detections of forest musk deer and 109 valid detections of alpine musk deer were retained for analysis (Figure 2). Temporal niche partitioning was examined within a seasonal framework specific to the climatic conditions of the reserve, classifying records into dry season (1 October–31 March) and wet season (1 April–30 September) [25].

Daily activity rhythms and temporal overlap between species were analyzed using kernel density estimation implemented in overlap packages in R v4.1.3 [26]. Temporal niche overlap was quantified by the coefficient of overlap (Dhat), ranging from 0 to 1, with higher values indicating greater overlap. Following sample size recommendations, Dhat1 was applied for comparisons involving fewer than 75 observations and Dhat4 for comparisons with 75 or more observations [27]. Activity pattern curves and overlap statistics were generated using the overlap and activity packages, allowing formal comparisons of diel activity and seasonal variation between the two species.

## 3. Results

### 3.1. Spatial Distribution Patterns

During the survey period, 493 infrared camera stations were deployed across Gongga Mountain National Nature Reserve. Results indicated that *M. berezovskii* was detected at 38 sites across an elevational range of 2723 to 4572 m ASL, with 428 photographic and 79 video captures, corresponding to 100 independent effective detection events. Furthermore, *M. chrysogaster* was recorded at 36 sites across an elevational range of 2870 to 4572 m ASL, yielding 344 photos and 103 videos, corresponding to 112 independent effective detection events. Co-occurrence was observed at 10 sites, all within the 2870–4572 m elevational range (Figure 1).

**Figure 3:**
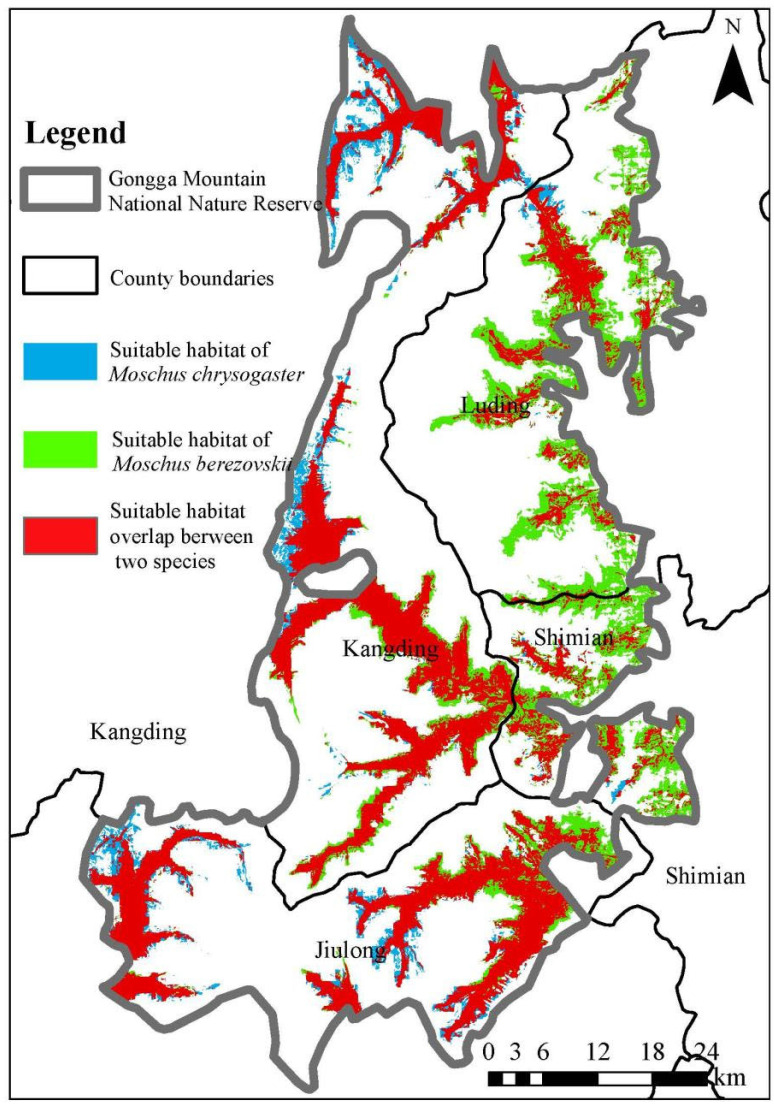
Overlap of suitable habitat for *Moschus berezovskii* and *Moschus chrysogaster* in Gongga Mountain National Nature Reserve.

Species distribution modeling identified distinct habitat suitability thresholds (TPT = 0.039 for *M. berezovskii*, 0.0364 for *M. chrysogaster*), coupled with a high degree of ecological niche overlap (Hellinger’s I = 0.905), reflecting strong congruence in environmental requirements. Within the 4091.4 km^2^ reserve, suitable habitat was estimated to cover 1195.91 km^2^ (29.23% of the reserve) for *M. berezovskii* and 903.9 km^2^ (22.09% of the reserve) for *M. chrysogaster*, with an overlapping area of 779.62 km^2^ (19.10% of the reserve). This shared area is primarily distributed across southern low-elevation valleys, the central Zimei–Jiebei corridors, the northwestern Laoyulin Valley, and the southeastern Hailuo–Yanzi valleys. Beyond the shared regions, *M. berezovskii* occupied an additional 416.29 km^2^ of suitable habitat in peripheral areas (Figure 3).

### 3.2. Correlation Between Habitat Suitability and Environmental Variables

Analysis of environmental contributions within the MaxEnt framework revealed a consistent hierarchy of influence on habitat suitability for both species. Vegetation exerted the strongest influence, accounting for 37.5% of habitat suitability for forest musk deer and 35.6% for alpine musk deer. Climate ranked second, with contributions of 28% and 34.5%, respectively. Topography, anthropogenic disturbance, and water sources each contribute less than 25% to model performance for both species, suggesting comparatively minor roles in shaping habitat distribution (Table 2).

At the individual variable level, elevation (20.3%), vegetation type (19.8%), and net primary production (17.7%) emerged as primary determinants for forest musk deer habitat suitability, while net primary production (21%), mean diurnal temperature range (16.5%), distance to water source (15.2%), and vegetation type (14.6%) were identified as major predictors in the alpine musk deer (Table 2).

**Figure 4:**
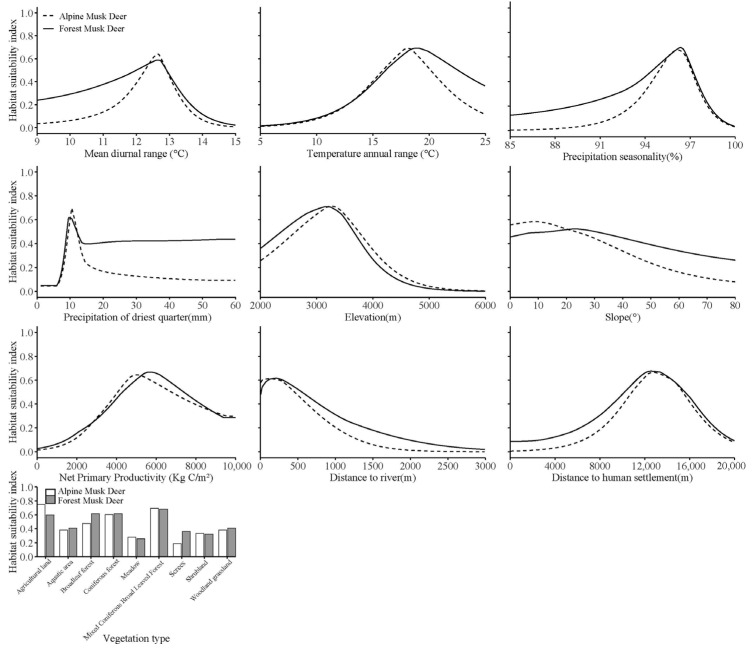
Response curves of habitat suitability for *Moschus berezovskii* and *Moschus chrysogaster* to environmental variables.

Both species exhibited remarkably similar ecological requirements, favoring environments characterized by a mean daily temperature range of 12–13 °C, elevations between 2500 and 3800 m, net primary productivity levels of 4000–6500 kg C/m^2^, a coefficient of variation in precipitation seasonality ranging from 95% to 97%, and dominance of coniferous or mixed conifer–broadleaf forests. The high degree of similarity in habitat preferences emphasizes their strong dependence on specific environmental conditions. Notably, examination of habitat suitability response curves indicated that *M. berezovskii* consistently occupied slightly broader or equivalent optimal ranges relative to *M. chrysogaster*, suggesting a narrower ecological tolerance and potentially higher sensitivity to habitat changes in *M. chrysogaster* (Figure 4).

### 3.3. Temporal Distribution Patterns

**Figure 5:**
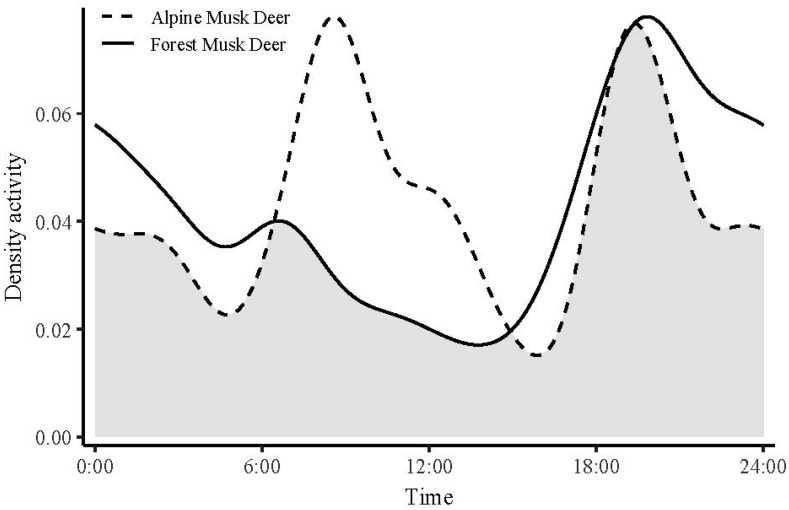
Daily activity patterns of *Moschus berezovskii* and *Moschus chrysogaster* across an annual time scale. Note: Overlap is represented by the shaded gray area.

At the annual scale, *M. berezovskii* and *M. chrysogaster* exhibited distinct diel activity patterns. Notably, *M. berezovskii* was predominantly nocturnal, with peak activity occurring between 18:00 and 02:00, and a secondary, smaller peak occurring between 05:30 and 07:30. In contrast, *M. chrysogaster* showed a bimodal activity pattern, with peaks between 08:00 and 10:00 and 19:00 and 21:00, and relatively lower nocturnal activity intensity (Figure 5).

**Figure 6:**
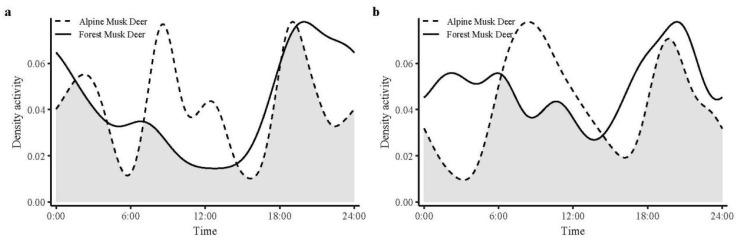
Daily activity patterns of *Moschus berezovskii* and *Moschus chrysogaster* in dry (**a**) and wet seasons (**b**). Note: Overlap is represented by the shaded gray area.

Seasonal analysis revealed consistent but seasonally modulated activity patterns relative to annual trends (Figure 6). During the dry season, *M. berezovskii* retained its strong nocturnal preference, with primary peaks between 18:00 and 02:00, and a secondary peak at 05:00–07:30. Conversely, *M. chrysogaster* exhibited more complex dry season behavior, characterized by four distinct peaks between 02:00 and 04:00, 08:30 and 10:30, 11:30 and 13:30, and 18:30 and 20:30. In the wet season, *M. berezovskii* showed multiple activity intensity peaks, with a pronounced and extended peak occurring between 18:00 and 22:00, showing significantly higher nocturnal than diurnal activity. In contrast, *M. chrysogaster* presented two peaks, occurring between 07:30 and 09:30 and 19:00 and 22:00 in the wet season, with morning activity surpassing evening activity in both intensity and duration. Temporal niche overlap, quantified by Dhat1, was 0.764 in the dry season and 0.792 in the wet season, indicating substantial but incomplete overlap between the two species.

## 4. Discussion

### 4.1. Spatial Ecological Niche

Among the three primary niche dimensions, habitat differentiation is regarded as the most fundamental and prevalent mechanism facilitating coexistence among closely related sympatric species [2], forming the basis for subsequent temporal and trophic segregation. Accurate identification of suitable habitat distribution, therefore, remains essential for effective wildlife conservation and management [28]. In Gongga Mountain National Nature Reserve, *M. berezovskii* and *M. chrysogaster* were found to occupy 1195.91 km^2^ (29.23% of the reserve) and 903.9 km^2^ (22.09% of the reserve), respectively. Both species exhibited fragmented and patchy distribution patterns, with core habitats concentrated in southern, central-southern, southeastern, and northwestern sectors of the reserve. Notably, a substantial spatial overlap of 779.62 km^2^ was identified, accounting for 65.19% of the suitable habitat for *M. berezovskii* and 86.25% of the suitable habitat for *M. chrysogaster*. Comparative analyses demonstrated that *M. berezovskii* maintained a larger extent of non-overlapping suitable habitat relative to *M. chrysogaster*, suggesting a broader ecological amplitude along the habitat niche axis. Environmental determinants, including precipitation seasonality, vegetation type, and net primary productivity, were shared drivers of habitat selection for both species. However, response curve analyses indicated that *M. berezovskii* generally occupied broader or comparable optimal ranges for key environmental variables, supporting the inference of greater environmental adaptability.

Unexpectedly, no significant elevational segregation was detected between the two species within Gongga Mountain. Although alpine musk deer have traditionally been regarded as high-altitude specialists (Wu et al., 2006) and forest musk deer as occupying lower elevations [7,29], both species exhibited overlapping elevational distributions in this system. This pattern may reflect an upward shift in treeline driven by regional climate conditions, altering habitat availability across altitudinal gradients. A similar context-dependent pattern has been reported in North American cervids: sympatric mule deer and white-tailed deer exhibit clear spatial segregation in heterogeneous mountainous landscapes, yet show substantial overlap in homogeneous agricultural regions [30,31]. These findings highlight the importance of considering geographical variation in habitat adaptability when designing conservation strategies.

### 4.2. Temporal Ecological Niche

Both *M. berezovskii* and *M. chrysogaster* exhibited bimodal activity peaks across annual and seasonal timescales, consistent with the crepuscular activity patterns observed in many ungulate species [32,33]. Despite this shared framework, pronounced interspecific differences emerged in the timing and intensity of daily activity rhythms. The results indicated that *M. chrysogaster* displayed a strong diurnal bias, with daytime activity levels markedly exceeding nocturnal activity, a pattern consistent with observations from the Qilian Mountains [34] and Upper Nujiang region [35]. In contrast, *M. berezovskii* exhibited a clear nocturnal profile within Gongga Mountain National Nature Reserve, with higher activity frequencies during nighttime hours, corroborating previous findings from Tangjiahe [36] and Daxiangling reserves [35]. Evidence from other regions further suggests considerable flexibility in the temporal activity patterns of *M. berezovskii*. In Hupingshan National Nature Reserve, forest musk deer did not exhibit marked diurnal or nocturnal tendencies [37], indicating that activity rhythms may vary in response to altitude, climatic conditions, and interspecific interactions. Similar patterns have been observed in other mammals, such as the serow and the Russian desman [38,39]. This behavioral flexibility results in a high degree of ecological plasticity and adaptive potential in response to varying environmental pressures.

Kernel density estimation further revealed moderate levels of temporal segregation between the two species. The annual diel activity overlap index was 0.792, with seasonal overlap indices of 0.764 in the dry season and 0.769 in the wet season. These relatively low overlap values suggest substantial temporal niche differentiation. Temporal partitioning likely represents a behavioral strategy to mitigate interspecific competition where spatial overlap is extensive, consistent with the hypothesis by Schoener (1974) that temporal niche differentiation can serve as a critical axis of competition among sympatric congeners [2].

### 4.3. Coexistence Mechanisms

Classical ecological theory posits that closely related species occupying the same geographic area cannot maintain stable coexistence if they exhibit substantial overlap across all ecological niche dimensions [40]. MacArthur (1967) [41] and subsequent studies emphasized that differentiation along at least one niche axis (spatial, temporal, or trophic) is essential to reduce interspecific competition and support long-term coexistence [42]. In the present study, *M. berezovskii* and *M. chrysogaster* exhibited a high degree of spatial niche overlap, particularly in elevational range and vegetation preferences, suggesting potential competition for similar environmental resources. According to the framework proposed by Kronfeld-Schor et al. (2003) [43], such overlap is indicative of exploitative competition, where species indirectly compete through shared resource consumption rather than direct interference. However, despite substantial spatial overlap, pronounced temporal differentiation was observed, likely serving as a key mechanism facilitating coexistence. Infrared camera-trap data revealed that both species frequently occupied the same sites but exhibited distinct activity periods, supporting the notion that temporal segregation effectively mitigates direct competitive interactions. Similarly, a study in Fanjingshan National Nature Reserve, Guizhou Province, found that four sympatric ungulates (*Elaphodus cephalophus*, *Rusa unicolor, Sus scrofa*, *Budorcas taxicolor*) displayed significant differences in temporal activity patterns, with variations across diel periods, seasons, and reproductive stages, further underscoring the importance of temporal niche partitioning in maintaining their coexistence [44]. From a broader biogeographical perspective, *M. berezovskii* demonstrates wider distribution and higher ecological adaptability, inhabiting diverse environments from the Qinling Mountains and southwestern highlands to the hilly regions of eastern China. In contrast, *M. chrysogaster* is primarily restricted to high-elevation areas of the Qinghai–Tibet Plateau and peripheral regions, reflecting a narrower ecological niche breadth [29]. These differences suggest that *M. berezovskii* possesses greater ecological plasticity and behavioral flexibility, enabling stronger resilience to environmental variation and competitive pressures [45].

Current population assessments indicate that both species maintain relatively low Relative Abundance Indices (RAI) within the Gongga Mountain National Nature Reserve [15], suggesting that population densities are not sufficiently high to exert mutual suppressive effects. Consequently, interspecific competition likely remains at the level of exploitative interactions rather than direct interference [46]. The environment of Gongga Mountain, characterized by minimal anthropogenic disturbance, high vegetation complexity, and abundant food, further promotes coexistence by reducing competition intensity. Additionally, the generalist browsing behavior of both species, combined with dietary flexibility in response to seasonal changes and plant community dynamics [6,47], contributes to further alleviation of resource overlap. In summary, the combination of marked temporal niche segregation, moderate population densities, and access to resource-rich, structurally complex habitats provides a robust ecological basis for the stable coexistence of *M. berezovskii* and *M. chrysogaster* within the Gongga Mountain region.

It is important to note, however, that the coexistence of these species may also be influenced by local predators and other herbivores. Due to data constraints, this study did not examine such biotic interactions, which warrant further investigation in future research to fully elucidate the mechanisms underlying sympatric coexistence.

## 5. Conclusions

Previous studies have widely posited that altitudinal distribution differences constitute the primary coexistence mechanism for forest musk deer (*Moschus berezovskii*) and alpine musk deer (*M. chrysogaster*). However, our analysis of decade-long data (2012–2021) from 493 infrared cameras in Gongga Mountain National Nature Reserve demonstrates that within the tree line elevation zone (reaching 3830 m), the two species exhibit no significant divergence in altitudinal distribution range (2870–4572 m). Strikingly, temporal partitioning between the two species is profoundly pronounced: *M. berezovskii* displays predominantly nocturnal activity while *M. chrysogaster* follows a distinctly diurnal pattern. These findings collectively suggest that species coexistence strategies may dynamically adapt to rapid environmental shifts in climate and vegetation. Consequently, conservation frameworks must explicitly account for species-specific adaptations to local climatic and phytogeographical conditions.

## Figures and Tables

**Table 1:** Ten environmental variables in MaxEnt model prediction.

Environmental Classification	Environmental Variable	Data Type	Source
Climate	Mean diurnal range (°C)	Continuous variable	https://worldclim.org
	Temperature annual range (°C)	Continuous variable	
	Precipitation seasonality (%)	Continuous variable	
	Precipitation of driest quarter (mm)	Continuous variable	
Topographic	Elevation (m)	Continuous variable	http://www.gscloud.cn
	Slope (°)	Continuous variable	
Vegetation	Net primary production(kg C/m^2^)	Continuous variable	http://loess.geodata.cn
	Vegetation type	Categorical variable	https://data.ess.tsinghua.edu.cn
Water source	Distance to river (m)	Continuous variable	http://loess.geodata.cn
Human disturbance	Distance to human settlement (m)	Continuous variable	

Note: Nine vegetation types were defined: agricultural land, aquatic area, broadleaf forest, coniferous forest, meadow, mixed coniferous–broadleaf forest, scree, shrubland, and woodland grassland.

**Table 2:** Contribution rate of environment variables for *Moschus berezovskii* and *Moschus chrysogaster*.

Environmental Classification	Environmental Variable	*Moschus berezovskii*	*Moschus chrysogaster*
Climate	Mean diurnal range (°C)	12.7	16.5
	Temperature annual range (°C)	0.3	3
	Precipitation seasonality (%)	11.1	10.7
	Precipitation of driest quarter (mm)	3.9	4.3
Topographic	Elevation (m)	20.3	6.2
	Slope (°)	1.7	5.9
Vegetation	Net primary production(kg C/m^2^)	17.7	21
	Vegetation type	19.8	14.6
Water source	Distance to river (m)	10.3	15.2
Human disturbance	Distance to human settlement (m)	2.1	2.4

## Data Availability

The data presented in this study are available upon request from the corresponding author.
